# Exploring the relationships between psychological variables and loot box engagement, part 2: exploratory analyses of complex relationships

**DOI:** 10.1098/rsos.231046

**Published:** 2024-01-03

**Authors:** Stuart Gordon Spicer, James Close, Laura Louise Nicklin, Maria Uther, Ben Whalley, Chris Fullwood, Jonathan Parke, Joanne Lloyd, Helen Lloyd

**Affiliations:** ^1^ Community and Primary Care Research Group (CPCRG), ITTC Building, Davy Road, Plymouth Science Park, Derriford, Plymouth PL6 8BX, UK; ^2^ Peninsula Medical School (Faculty of Health), University of Plymouth, Plymouth Devon PL4 8AA, UK; ^3^ School of Psychology, University of Plymouth, Plymouth Devon PL4 8AA, UK; ^4^ School of Education, Faculty of Education, Health and Wellbeing, University of Wolverhampton, Wolverhampton WS1 3BD, UK; ^5^ Enterprise and Innovation, Faculty of Health, Education and Life Sciences, Birmingham City University, Seacole Building, Edgbaston Campus, Birmingham, UK; ^6^ School of Natural, Sport and Social Sciences, University of Gloucestershire, Cheltenham, UK; ^7^ Director, Sophro Ltd, Newark Beacon, Newark, UK; ^8^ Cyberpsychology Research Group, School of Psychology, Faculty of Education, Health and Wellbeing, University of Wolverhampton, Wolverhampton WV1 1LY, UK

**Keywords:** loot boxes, video gaming, gambling, digital harms, addictive behaviours, wellbeing

## Abstract

In a pre-registered survey linked to this paper (Exploring the relationships between psychological variables and loot box engagement, part 1: pre-registered hypotheses), we confirmed bivariate associations between engagement with loot boxes (purchasable randomized rewards in video games) and measures of problem gambling, problem video gaming, impulsivity, gambling cognitions, experiences of game-related ‘flow’, psychological distress and reduced wellbeing. However, these variables have complex relationships, so to gain further insights, we analysed the dataset (1495 gamers who purchase loot boxes and 1223 purchasers of non-randomized content) in a series of Bayesian mixed-effects multiple regressions with a zero-inflation component. The results challenge some well-established results in the literature, including associations between loot box engagement and problematic gambling measures, instead suggesting that this relationship might be underpinned by shared variance with problem video gaming and gambling-related cognitions. An entirely novel discovery revealed a complex interaction between experiences of flow and loot box engagement. Distress and wellbeing are both (somewhat contradictorily) predictive of participants engaging with loot boxes, but neither correlate with increasing loot box risky engagement/spend (among those who engage). Our findings unravel some of the nuances underpinning loot box engagement, yet remain consistent with narratives that policy action on loot boxes will have benefits for harm minimization.

## 1. Introduction

Loot boxes are purchasable items in video games, with randomized content that varies in financial and psychological value [1,2]. They are available in the majority of games across different formats, including console games, PC games and mobile games, and are often available to children [1,3]. Evidence from systematic reviews and meta-analyses has established robust associations between loot box engagement, and measures of both problem gambling and problem video gaming [4–8]. Similarly, associations have also been investigated between loot box engagement and a range of psychological variables, including impulsivity (where results are equivocal [9–13]), gambling cognitions (evidence is limited [14]), and psychological distress and wellbeing (results again equivocal [15–17]). Other variables, such as game-related experiences of ‘flow’ remain unstudied in the context of loot boxes, but have been linked with problematic video gaming [18]. To investigate such observations, we conducted a pre-registered survey of 1495 gamers who purchase loot boxes (henceforth, ‘LB cohort’) and 1223 gamers who purchase other, non-randomized game content (henceforth, ‘nLB cohort’), first investigating these relationships with a series of pre-registered bivariate analyses on this data that are reported in a linked publication, ‘Exploring the relationships between psychological variables and loot box engagement, part 1: pre-registered hypotheses' [19].

Our findings confirmed previously established relationships between loot box engagement, and both PGSI scores (i.e. a measure of problem gambling) and problem video gaming. These effects were found both with self-reported loot box spend and a validated measure of risky loot box engagement—the RLI [14]. This validated measure captures how gamers feel about their engagement, for example by asking them to rate their agreement with statements including ‘once I open a loot box, I often feel compelled to open another’—and has been used in several previous studies [14,16,20,21]. This measure therefore contrasts with self-reported loot box spend, in that it may be a more direct measure of risky behaviour. Gambling-related cognitions—which are robustly associated with symptoms of problematic gambling [22]—were also positively correlated with both risky loot box engagement and loot box spend, consistent with previous observations [14]. Our study is, to our knowledge, also the first to establish links between loot box engagement and experiences of game-related flow. Previous studies have linked flow with problematic video gaming [18] and with PGSI status of participants in a laboratory-based slot machine study [23], and the relationship between this state of high absorption and (potentially problematic) loot box engagement further emphasizes the shared psychological components of loot boxes and traditional forms of gambling.

While the majority of our pre-registered hypotheses were supported, the results suggested complex relationships between some of the psychological variables. For example, impulsivity revealed a positive correlation with risky loot box engagement, but not loot box spend; providing a possible explanation of previous equivocal results [9,10], where impulsivity might be specifically linked with risky types of loot box engagement. It is also possible that risky loot box engagement is a more reliable measure than loot box spend, as it requires general estimations of whether certain behaviours and motivations have featured in one's purchasing, rather than relying on self-report estimations of objective continuous measures, which are sometimes prone to inaccuracies [24,25].

Only a limited number of previous studies have specifically investigated how loot box engagement might influence player wellbeing, with mixed results and evidence of possibly indirect effects [15–17]; including one study that concluded both positive and negative (past week) moods might be associated with higher loot box spend [16]. Our bivariate analyses found no evidence of a relationship between loot box spend, and either wellbeing or psychological distress. However, we did find evidence of a relationship between risky loot box engagement and both of these variables—suggesting a difference in the variables that influence spend versus those that specifically influence risky engagement.

With demographic variables, our bivariate analyses [19] established that male sex was predictive of loot box spend (consistent with previous observations [26]), but not risky loot box engagement (see previous paper for discussion [19]). Conversely, younger age was predictive of risky loot box engagement, but not loot box spend (possibly owing to the lower disposable income of younger gamers). Income was predictive of neither risky loot box engagement nor loot box spend, also consistent with earlier findings [27].

Following our pre-registered analysis plan [28], relationships between the above psychological variables and loot box engagement were first investigated on a bivariate, one-by-one basis [19]. However, the relationships between these various overlapping constructs (i.e. impulsivity, problematic gaming, problematic gambling, gambling cognitions, etc.) are likely to be complex and multidirectional [29]. While much previous loot box research has used bivariate correlations, several studies have employed more complex analyses. These include a path analysis of the relationship between loot box spend, and measures of both problem gambling and problem video gaming [15]; a hierarchical regression of risky loot box engagement and gambling-related variables [14]; and a multi-variable regression [11] of problem gambling and loot box spend, after controlling for impulsivity and demographic variables. Our study similarly employs a more complex analytical approach, but using a larger set of variables than these previous studies. These analyses were exploratory, but again covered in our pre-registration plans (i.e. we pre-registered the methodology, but made no hypotheses). We used Bayesian mixed-effects regressions to understand how the variables from our bivariate analyses operate when analysed within a single model. We were particularly interested in understanding whether the bivariate relationships would be maintained using this multiple-predictor approach—and which variables would be most predictive.

### 1.1. The present study

We conducted a set of Bayesian mixed-effects multiple regressions with a zero-inflation (ZI) component [30]. Such models account for both the LB (loot box) cohort and the nLB (non-loot box) cohort within a single model, enabling investigation of differences both within and between the two cohorts. Effectively, this type of regression combines both a ZI regression and a linear regression into a single model; the former comparing the two cohorts, and the latter assessing relationships within the LB cohort only. This method therefore shows which variables are predictive of engagement/spend on loot boxes versus no engagement/spend in the ZI component, and which variables correlate with increasingly risky loot box engagement/increasing spend among gamers who already engage with loot boxes in the linear component.

## 2. Methods: design and measures

Details about data collection, measurement instruments and data exclusions are provided in our previous paper [19]. This paper uses the same dataset. However, in brief, our study design was pre-registered on the Open Science Foundation [28]. Our cohort included data from a sample of both loot box (LB cohort; *n* = 1495) and non-loot box purchasing (nLB cohort, *n* = 1223) gamers. Participants were recruited from the survey recruitment platform, Prolific [31], resided in the UK, were adults (18+), and had been previously identified (via a short screening survey [26]) as videogame players who purchased game-related content. Data were collected from 8 March 2021 to 24 March 2021 on the Qualtrics [32] survey platform. Data from 91 participants were removed, with missing data on monthly income and/or sex, to ensure complete data for all participants were included. This was so that all the predictor variables were numeric for running the analyses. Consistent with any two-level categorical variables, sex was treated by the model as a numeric binary (i.e. 1's and 0's).

### 2.1. Survey measures and instruments

The following are the variables relevant to the analyses in the paper (additional variables were collected, but only used in the previous paper [19]). Our two outcome measures (of loot box engagement) were: typical monthly spend; and the risky loot box index (RLI), a tool that measures risky or problematic loot box engagement. The RLI was our primary outcome measure. We collected demographic variables of age and gender, along with self-reported income. Our study included a range of previously validated measurement instruments (box 1).

Box 1. **Measurement instruments included in survey. Throughout this paper, these are usually referred to by the ‘concept,’ rather than the instrument or abbreviated instrument name (abbr.).**

**Table:** 

concept	abbr.	instrument and reference
risky loot box engagement	RLI	risky loot box index [14]
problem gambling	PGSI	problem gambling severity index [33]
problem video gaming	IGD	internet gaming disorder short form [34]
gambling cognitions	GCRS	the gambling-related cognition scale [35].
impulsivity	BIS-Brief	Barratt impulsiveness scale (bis-brief) [36], 8-item version [37].
flow	GES-Flow	previously used 5-item set of the game experience questionnaire [23,38]
wellbeing	WEMWBS	The Warwick–Edinburgh mental wellbeing scale (WEMWBS) [39].
psychological distress	K-10	Kessler psychological distress (K-10) [40]

### 2.2. Data processing

All data processing and analysis was conducted in R [41]. For instrument scoring, we used standard, published methods of calculation. We rescaled all responses to start at zero [42] for consistency; a minor adjustment that will not influence the results. Details about the analytical methods are provided in the next section.

### 2.3. Methods: Bayesian mixed-effects regressions

To explore relationships between our variables—and thus determine which variables are most predictive of loot box engagement once all variables (problem gambling score, problem video gaming, gambling cognitions, impulsivity, wellbeing, psychological distress, flow, sex, age and income) are considered together—we ran three Bayesian mixed-effects multiple regressions with a ZI component. Each regression had a different outcome variable; RLI, loot box spend and loot box spend adjusted for total income. In fitting models with multiple predictors, we were interested in characterizing which variables were most predictive, and which variables would remain predictive compared to single predictor models/bivariate approaches.

The ZI component of the model assesses which variables are most predictive of participants *engaging* versus *not engaging* with loot boxes (i.e. testing for a difference between the LB and nLB cohorts), while the linear component assesses which variables are most predictive of increasing engagement among loot box purchasers (i.e. establishing which variables are most significantly correlated with continuous measures of engagement, within the LB cohort). We report the effects of predictors within both parts of the model. Additionally, the regressions used a negative-binomial response distribution, to account for the large number of participants with a loot box spend/RLI score of zero (i.e. the nLB cohort of participants).

We ran two types of Bayesian ZI mixed-effects negative-binomial multiple regressions for each of the outcome variables. The first was a single predictor model, in which each variable was analysed on its own, as a predictor of the outcome (similar to conducting a bivariate correlation). The second was a multiple regression, in which all predictors were analysed together. We used a horseshoe prior (implemented in Stan [43]), to provide a form of penalization [44], using L1-normalization to ‘shrink’ the predictive power of variables. This enables variable selection—where some variables can be accepted and others can be dismissed. We originally intended to penalize using both Lasso and Horseshoe priors. However, we ultimately chose the stricter Horseshoe prior only, as the Lasso had a negligible effect on the predictors (a consequence of having such a large dataset). Our methods were selected because traditional variable selection procedures (e.g. stepwise regression/forward selection) are known to perform poorly as an exploratory analysis, given multiple correlated predictors [45].

We specified the following predictor variables in each model. *Risky loot box engagement (RLI) model:* problem gambling (PGSI), problem video gaming (IGD), gambling-related cognitions (GRCS), impulsivity (BIS-Brief), flow (GES-Flow), wellbeing (WEMWBS), psychological distress (K-10), sex, age, income. *Loot box typical monthly spend model:* problem gambling (PGSI), problem video gaming (IGD), gambling-related cognitions (GRCS), impulsivity (BIS-Brief), flow (GES-Flow), wellbeing (WEMWBS), psychological distress (K-10), sex, age, income.

While we did initially attempt a third regression with the adjusted loot box spend model (i.e. adjusted to income), this is not reported (see later section ‘Exploratory analyses: deviation from pre-registration document’).

For all models, we employed standard techniques to check for model convergence (visual inspection of Markov chain Monte Carlo (MCMC) trace-plots, Rhat values, and effective sample size (ESS) values). Posterior distributions for parameter estimates are summarized and presented graphically (mean values with 95% high dimensional credible intervals (HDCI)). For the ZI component of the regression models, if the 95% HDCI's were discrete from an odds ratio of 1, this was interpreted as substantial evidence of a variable predicting loot box engagement (between loot box purchases and non-loot box purchasers). Distributions greater than 1 were interpreted as positively predictive, while those less than 1 were interpreted as negatively predictive. Similarly, for the linear component of the models, if the 95% HDCI's were discrete from an RLI score/£ spend of 0, this was interpreted as substantial evidence of a variable predicting increasing loot box engagement (within loot box purchasers). Distributions greater than 0 were interpreted as positively predictive, while those less than 0 were interpreted as negatively predictive.

We conducted two additional analyses (i.e. non-pre-registered), to further interpret the results of Bayesian regressions. These both used model averaging. The first was to assess which predictor variables are the most ‘labile’ across multiple combinations of models—i.e. how much they vary in predicting the outcome variable. Owing to computational power limitations, we used a frequentist (rather than Bayesian) implementation of the ZI multiple regressions (implemented in the Dredge package in R). We conducted model averaging across every possible combination of predictors (up to a maximum of seven; also owing to limitations in computational power). The lability of variables was assessed by plotting 95% HDCI. The second additional analysis was an extension of the above model averaging procedure, to assess which predictors were most ‘disruptive’ of other predictors—i.e. how much each predictor changed the predictiveness of other predictors across multiple combinations of models (again up to a maximum of seven). This was assessed by plotting the effect of each predictor on other included predictors.

## 3. Results

### 3.1. Introduction to regression model results

**Figure 1.**
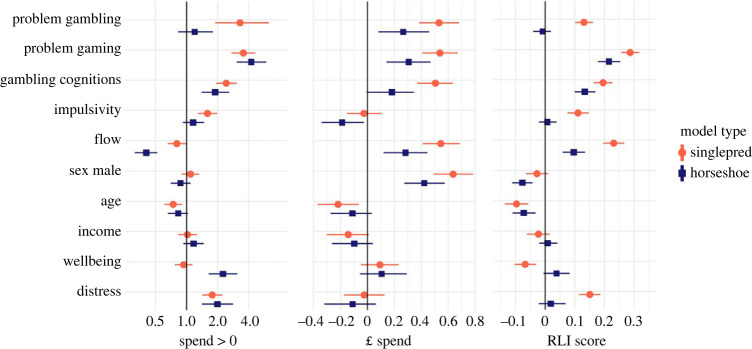
Results of Bayesian exploratory regressions with loot box spend and risky loot box engagement as outcomes. The zero-inflation component is presented in the left-hand panel (‘spend > 0’: odds ratio of engagement). This shows how predictive each variable is of participants *spending* versus *not spending* on loot boxes. This measure is only shown for loot box spend, because RLI results effectively show the same information (whether participants are interacting with loot boxes in some way or not)—and spending versus non-spending is intuitively easier to interpret than risky engagement versus non-engagement. However, the full results are available in the supplementary materials at https://osf.io/gh634/. Odds ratios to the right of the vertical line (at equal odds ratio of 1.0) are more predictive of spending versus not spending (i.e. spend greater than zero), while those to the left are preventative of spending. The linear component for spend is presented in the middle panel (‘£ spent’: coefficient of increasing spend), and the linear component for risky loot box engagement is presented in the right-hand panel (‘RLI score’: coefficient of risky engagement). These show how predictive each variable is of participants (who already engage with loot boxes) spending more/engaging in an increasingly risky manner. Coefficients to the right of the vertical line are more predictive of increasing spend/increasingly risky engagement, while those to the left are predictive of lower spend/less risky engagement. Results of both the single predictor (singlepred, in red) models (a separate model for each variable), and the multiple-predictor (with horseshoe prior, blue) model, are presented. For all models across both panels, the horizontal lines represent 95% HDCI. Distributions that are discrete from the black horizontal lines can be interpreted as providing evidence of an effect. Note that male sex is coded as 1 (i.e. to the right).

The data and R code for running the models are openly available at https://osf.io/gh634/. Below, we report results of Bayesian mixed-effects negative-binomial multiple regressions with a ZI component, for both loot box spend and RLI. As detailed in the methods, the ZI component of the model indicates which variables are predictive of participants *engaging/spending* versus *not engaging/spending* on loot boxes (i.e. comparison of loot box purchasers versus non-loot box purchasers). The linear component of the model indicates which variables are predictive of participants (who already use loot boxes) engaging in an increasingly risky manner (RLI model), or spending increasing amounts of money (LB spend model). For each outcome variable, the results of the ZI and linear components are presented side-by-side in figure 1.

With these models, it should be noted that there is an additional ZI component which is not reported in the body of this paper: the ZI component of the RLI model, which captures zero versus non-zero RLI. Nonetheless, these results are nearly identical with the ZI for spending versus not spending (see electronic supplementary material at https://osf.io/gh634/ for complete data, which notes any differences). Below, we only present the ZI model for spend data, as this is the most logical representation of loot box engagement versus non-engagement (i.e. participants with zero RLI may actually be engaging with loot boxes, but scoring zero on the RLI instrument).

### 3.2. Summary of results with single predictors

In our models, the single predictor results (i.e. single regression for each variable as a predictor of the outcome, red bars in figure 1) broadly replicate our previous findings from bivariate correlations, as would be expected [19]. They confirm that variables including problem gambling scores, problem video gaming, gambling-related cognitions and game-related experiences of flow were predictive of both *increasing* loot box spend and risky loot box engagement (linear components; middle- and right-hand panel of figure 1). Furthermore, higher impulsivity, higher distress and (in contrast with our bivariate result) lower wellbeing were predictive of increasingly risky loot box engagement. Monthly income was predictive of neither risky loot box engagement nor loot box spend—consistent with previous results of open access data [27].

A similar pattern of results was seen for single variables predicting participants spending versus not spending on loot boxes (left-hand side of figure 1). However, there were some discrepancies. For instance, flow did not predict participants spending versus not spending on loot boxes, but did predict increasingly risky engagement and spend, for participants already using loot boxes. A similar pattern was observed for wellbeing, which was not predictive of spending versus not spending—but was (negatively) predictive of increasingly risky engagement among the LB cohort. Male sex was not predictive of spending versus not spending^1^, or increasingly risky engagement, but was predictive of increasing spend. Distress was predictive of spending/engaging versus not spending/engaging only.

### 3.3. Summary of results with multiple regressions

Overall, the ZI components (figure 1, left panel, blue lines) indicated several variables predictive of spending versus not spending on loot boxes (i.e. a difference between the LB and nLB cohorts). This includes problem video gaming, gambling-related cognitions, distress and—seemingly counterintuitively—wellbeing (see later Discussion). Flow was negatively predictive of spending versus not spending (i.e. higher flow was protective), and younger age was not predictive of spending versus not spending.^2^ The linear components reveal several variables predictive of increasing loot box engagement and spend (figure 1, middle and right panels). For increasingly risky loot box engagement, the primary predictors were problem video gaming, gambling-related cognitions and game-related experiences of flow. This result with flow was in contrast to the negative association with spending versus not spending. There were also associations with *female* sex, and an inverse relationship with age. Neither wellbeing, distress nor income were predictive of increasingly risky engagement. For spend, there were positive associations with problem gambling scores, problem video gaming, flow (again in contrast to the negative association for spending versus not spending) and male sex. There was a marginal result for gambling-related cognitions, where the lower bound of the 95% HDCI was exactly zero. Neither wellbeing, distress nor income were predictive of increasing spend. Impulsivity had a negative association with increased spend but no relationship with increasingly risky engagement.

Perhaps most notably, the relationship between problem gambling scores and risky loot box engagement was not observed, and the relationship between problem gambling scores and loot box spend was only observed in the linear component of the multiple regression. Gambling-related cognitions remained predictive of risky loot box engagement.

While the contrasting result with flow might initially appear contradictory, these results suggest that once all other variables were controlled for, flow was protective against participants spending versus not spending on loot boxes, but once participants do start to engage flow becomes predictive of higher engagement (both spend and risky engagement).

Overall, these results produced some unexpected findings, particularly around problem gambling scores, flow, distress and wellbeing. Our findings are more easily understood when contextualized alongside our ‘lability analyses’, which enable an exploration of how each variable within the multiple regressions is influenced by the other variables. Below, we discuss the results of the lability analysis, before returning to a variable-by-variable synthesis of the results in the discussion.

### 3.4. Summary of results of lability and disruption analyses

**Figure 2.**
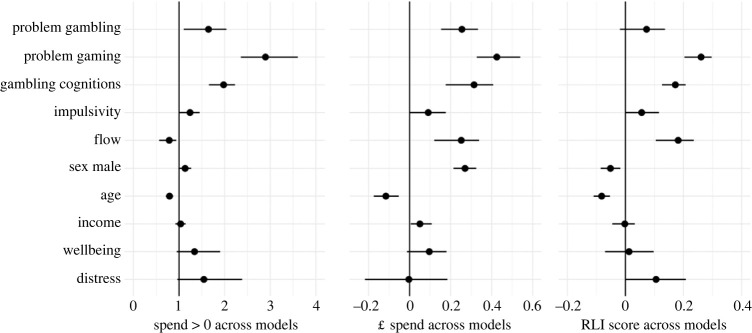
Results of lability analysis (model averaging) with both loot box spend and risky loot box engagement as outcomes. Consistent with figure 1, the left-hand side shows the zero-inflation component of the (spend) model, while the middle shows the linear component for spend, and the right-hand side shows the linear component for RLI. The points indicate the mean odds ratio/coefficient across all models, and the lines represent the distribution across all models (95% HDCI); i.e. longer lines indicate a variable that is more labile across different models. Similar to figure 1, the spend and RLI zero-inflation results were very similar, so only spend is included in this figure (full results at https://osf.io/gh634/).

As described in the Methods, we performed an additional ‘lability analysis’ to aid interpretation of the regressions and clarify differences between the single- and multiple-predictor models. This established how much each variable changes in predictiveness, as different combinations of variables are added into the model, enabling insights into which variables were most stable. This was conducted with a complementary ‘disruption analysis’, which established how much each variable alters the predictiveness of other variables as different combinations are added into the model. Results of the lability analysis are reported in figure 2.

As the aim of this analysis was to determine the lability of each predictor (i.e. as different combinations of variables are added), the important result is not the regression coefficients/odds ratios themselves (i.e. the dots), but the width of the confidence intervals for each variable (i.e. the line width). These intervals represent the amount of variability (i.e. lability) for each predictor across all model combinations; i.e. a wider line indicates a variable that is more labile. In short, the Bayesian regressions tell us which variables are most predictive of the outcomes, and the lability analyses tell us which variables are most stable across different model combinations.

Here, the most stable variables were the demographic variables (age, sex and income). On the whole, the psychological instruments were more labile (an unsurprising result, given the potential relationships between the underlying constructs), although there was considerable variation. Wellbeing and psychological distress were among the most labile predictors, with a number of other variables also disrupting distress (especially problem video gaming). Problem gambling scores and problem video gaming were also comparatively labile. Flow, gambling cognitions and impulsivity generally had an intermediate lability compared to the other variables, although there was some variation between models and components.

**Figure 3.**
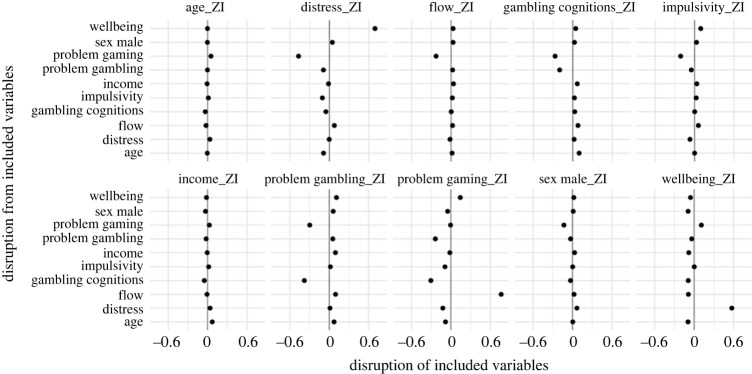
Results of disruption analysis (model averaging) with RLI as the outcome for zero-inflation (ZI) component. The points indicate the mean disruption across all models for each predictor. By reading across the horizontal axes for each predictor, the amount of disruption on each other predictor can be gauged. Similarly, by reading down the vertical axes for each predictor, the amount of disruption from each other predictor can be gauged. Disruption to the left means that variable is reduced in predictiveness, while disruption to the right means increased.

**Figure 4.**
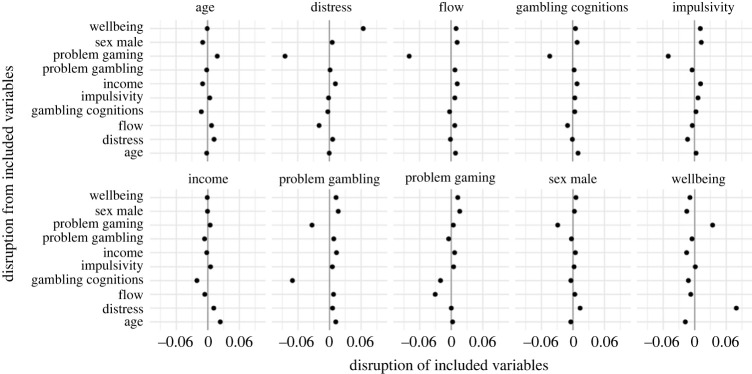
Results of disruption analysis (model averaging) with RLI as the outcome for linear component. The interpretation is the same as for figure 3.

**Figure 5.**
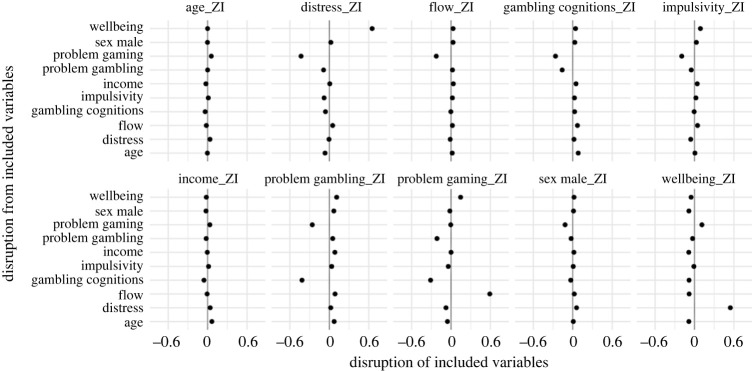
Results of disruption analysis (model averaging) with loot box spend as the outcome for zero-inflation (ZI) component. The interpretation is the same as for figures 3 and 4.

**Figure 6.**
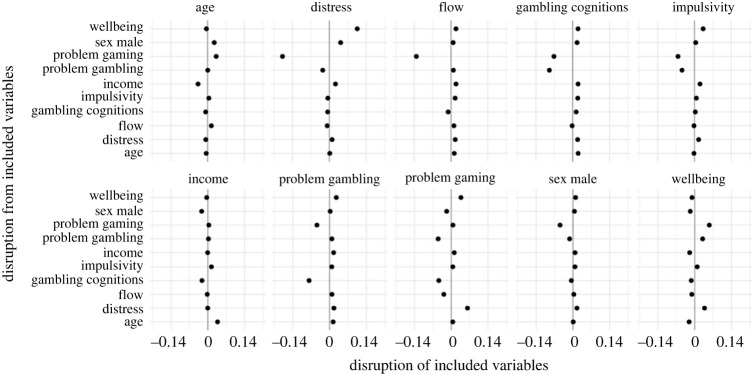
Results of disruption analysis (model averaging) with loot box spend as the outcome for linear component. The interpretation is the same as for figures 3–5.

The disruption analyses, reported in figures 3–6 further reveal the impact variables can have on each other when added to the models. Problem gambling scores were disrupted most by problem video gaming and (unsurprisingly) gambling-related cognitions. With problem video gaming, some of the largest disruption comes from impulsivity and flow. Problem video gaming was also disrupted by problem gambling scores. Disruption between problem gambling scores and problem video gaming is consistent with evidence of a relationship between these variables [8]. In addition to being among the most labile predictors, wellbeing and psychological distress were both (unsurprisingly) substantially disruptive of each other, with several other variables also disrupting distress (especially problem video gaming). The demographic variables, age, income and sex, received some of the smallest disruption from other variables. They were also not very disruptive of other variables, although there was some disruption between income and age—two variables that (as previously noted) are known to correlate [19].

There are some interesting observations within the results of the disruption analyses. For example, when risky loot box engagement was the outcome, gambling-related cognitions were disruptive of problem gambling scores, but problem gambling score was not disruptive of gambling-related cognitions. This suggests that gambling-related cognitions (i.e. GRCS measure) is capturing something unique (with respect to loot boxes) that problem gambling score (i.e. PGSI measure) does not. This may suggest that whatever component of the problem gambling score is predictive of risky loot box engagement is almost entirely captured within gambling-related cognitions. The results of the lability and disruption analyses are discussed in more detail in the discussion, variable-by-variable, in relation to the results of the Bayesian regressions and findings from previous loot box literature.

### 3.5. Deviations from pre-registered regressions

The analytic approach for the Bayesian regressions was pre-registered, albeit marked as a purely exploratory analysis. Nonetheless, we made a few necessary deviations from the pre-registration document. First, we did not include RLI as a predictor in the loot box spend models, as these variables are highly correlated—most notably, all participants with zero loot box spend will also have a zero score for RLI. The RAFFLE scale of loot box motivations [46] was not included as a predictor in any models for the same reason. The predictor variables were otherwise as specified.

We also pre-registered a model with ‘adjusted loot box spend’ (with loot box spend adjusted for self-report annual earnings). However, this model failed inspection measures, and is therefore not reported: the MCMC trace plots indicated that the models did not adequately converge, and the excessively wide HDCI's on the posterior distributions suggested that the model configuration was not adequately capturing the data. The ESS and Rhat values were acceptable, although the ESS values were lower than for the other two models. Therefore, the results do not provide an accurate measure of the relationship in the data. While the reasons for this are unclear, our previous pre-registered hypotheses were not met for adjusted spend anyway [19], so there were no findings to explore with the Bayesian regressions (making this third model somewhat redundant). However, the code for running these models is nonetheless included at https://osf.io/gh634/. The risky loot box engagement (RLI) and unadjusted loot box spend models passed all inspections.

As discussed in our pre-registration document, we conducted both normal prior and horseshoe prior (i.e. penalized) models (see Methods), which act as a form of sensitivity analysis, where we expected the results to be consistent across both models. Visual inspection of results confirmed this to be the case, indicating that results are robust, and therefore we present only results from the stricter penalized regression models. Full code is included within https://osf.io/gh634/.

## 4. Discussion

The single predictor regression models largely replicated our previous findings [19], revealing that several variables, when studied on a simple variable-by-variable analysis, are predictive of risky loot box engagement and/or loot box spend, including problem gambling score, problem video gaming, impulsivity and gambling-related cognitions. However, when included in multiple regression models, the results reveal a number of surprising observations, which on initial appraisal might appear counterintuitive or contradictory to previous literature. One of the most notable of these results is the lack of association between problem gambling score and risky loot box engagement. These results are more easily understood when contextualized alongside the lability analysis (how much each variable changes, according to the variables added into the model) and disruption analysis (i.e. how much each variable is influenced by each other variable); enabling discussion of *why* differences are observed between single- and multiple-predictor models. Below, these results are discussed variable-by-variable.

### 4.1. Problem gambling score

The results of the single predictor regressions are consistent with our previous pre-registered hypotheses [19], using simple bivariate correlations and numerous previous studies [4–8]. These show robust, moderately sized associations between problem gambling score and loot box engagement (measured by either spend or RLI). However, the results of our multiple regressions produce contrasting findings. Problem gambling score is no longer associated with participants *spending* versus *not spending* with loot boxes (i.e. loot box cohort versus non-loot box cohort). It is also not predictive of increasingly risky engagement, although it is still predictive (albeit at a reduced level) of increasing spend on loot boxes (where correlations with spend are the most commonly reported measurement of loot box engagement in the literature [4–8]).

While this result is somewhat surprising, the disruption analysis (figures 3–6) helps reveal why this association disappears in the multiple regression, where the difference between the models is primarily explained by disruption from problem video gaming and gambling-related cognitions. Such results are consistent with previous relationships, where it is known that there are associations between problem gambling score and problem video gaming [8], and also between problem gambling score and gambling-related cognitions in a video gaming context [47–49]. Importantly, problem video gaming and gambling-related cognitions reduce the predictiveness of problem gambling score for both the ZI and linear components of the model. In other words, problem gambling score becomes less predictive of loot box engagement once problem video gaming and gambling-related cognitions are controlled for. This could be a consequence of shared variance between these variables (i.e. they are partially measuring aspects of the same phenomena, entirely plausible for problem gambling score and gambling-related cognitions). Alternatively, these variables interact in some way—either as a direct variable interaction or indirectly via a pathway [15]. However, the vast number of potential models is beyond the scope of this exploratory study. Overall, our findings suggest that previously reported relationships between problem gambling score and loot box engagement might not be owing to direct effects, but instead are explained largely by a combination of shared cognitions about chance (e.g. that a series of losses, for example, mean that a ‘win is due’; previously established to be associated with problematic gambling behaviours [35,47–49]) and the shared variance between the psychologically related behaviours of problem video gaming and problem gambling score.

### 4.2. Gambling-related cognitions

In contrast to problem gambling score, gambling-related cognitions remained predictive (in multiple regressions) of both spending/engaging versus not spending/engaging on loot boxes and also increasingly risky loot box engagement (among those who already engage). Nonetheless, gambling cognitions were disrupted by both problem gambling score and problem video gaming according to the disruption analyses (although this variable was less labile than problem gambling score). However, problem gambling score had negligible impact on the predictiveness of gambling cognitions for increasingly risky engagement. This contrasts with the reverse situation, where gambling cognitions were highly disruptive of problem gambling score. This observation is consistent with the explanation that the well-established relationships between loot box engagement and measures of problem gambling, in part, may be mediated by underlying gambling cognitions. As a predictor of risky loot box engagement, problem gambling score predicts nothing additional beyond the gambling-related cognitions, although the asymmetrical disruption between these variables suggests that gambling cognitions capture something unique as a predictor of risky loot box engagement, which problem gambling score does not. However, gambling cognitions (as measured by the GRCS) captures ‘a perceived inability to stop gambling’, so both scales are arguably measuring problematic involvement in gambling. Such results shed light on what may be a primary mechanism underpinning the previously demonstrated association between problem gambling score and loot box engagement.

Interestingly, in another loot box survey that used multiple regression analyses [50], associations between loot box purchasing and problem gambling score remained after controlling for psychological variables (including impulsivity and emotion dysregulation). This study, however, did not include gambling-related cognitions—a key mediating variable within our results.

Taken together with problem gambling score, such results may indicate something further about the processes underlying the relationship between loot box engagement and gambling. It might be that upstream gambling cognitions (i.e. risk) are predictive of engaging/spending versus not engaging/spending, while downstream problem gambling score (i.e. harm) is predictive of increasing spend. Finally, it is worth emphasizing that our observations are not necessarily contradictory with other previous literature, which has primarily focused on relationships between problem gambling score and loot box spend; a positive association that is still observed within our multiple regressions.

### 4.3. Problem video gaming

In contrast to problem gambling score, problem video gaming was not substantially altered in the multiple-predictor models. Increased problematic video gaming remained predictive both of engaging/spending versus not engaging/spending on loot boxes, and of increasingly risky engagement/higher spend. Despite problem video gaming being somewhat labile, it remained consistently predictive of both outcome variables. There was notable disruption from several other variables, with gambling-related cognitions and flow being those most consistently disruptive across the various model components. However, other variables were also disruptive on some components, including distress, wellbeing and problem gambling score. This suggests that the relationship between problem video gaming and loot box engagement might be subject to indirect influence from several variables through their impact on problem video gaming. For instance, one study suggests that elevated distress is related to problem video gaming and problem gambling score, which is in turn indirectly related to loot box spending [15]. Further research is required to disentangle such causal pathways.

### 4.4. Impulsivity

Consistent with the results of our bivariate analyses, the single predictor models revealed that impulsivity had associations with spending/engaging versus not spending/engaging on loot boxes and with increasingly risky loot box involvement (among those who already engage) in, but not with increasing loot box spend. Such results would appear to suggest that impulsivity might be specifically linked with risky loot box engagement. However, the results of the multiple regressions suggest that impulsivity is not, in fact, predictive of risky loot box engagement, once the other variables have been controlled for.

The disruption analyses reveal possible explanations for this surprising result. Here, problem video gaming was consistently disruptive of impulsivity across all model components, and problem gambling score also disrupted impulsivity as a predictor of loot box spend. The most straightforward explanation is that impulsive behaviours relevant to loot boxes are largely mediated via problem video gaming, and impulsivity itself is left with nothing more to explain.

Again, this finding is also suggestive of a possible pathway, where impulsivity might indirectly influence loot box engagement via other gaming behaviours. Such a pathway would be consistent with previously well-established links between impulsivity and disordered gaming [51], and it is therefore possible that bivariate links between impulsivity and loot box engagement are an artefact of impulsivity's strong relationship with problem video gaming. However, a recent publication (after our own data collection window) highlighted the potential limitation in measuring impulsivity as a unidimensional construct [13]. This study found evidence of a relationship between loot box purchasing, and both positive urgency and sensation seeking, but no relationship with negative urgency and lack of premeditation—measures that are known to be linked with problem gambling score. Our unidimensional measure of impulsivity may therefore be unable to capture these potential complexities.

### 4.5. Flow

From all the variables, flow has perhaps the most nuanced relationship with loot box engagement. Multiple regressions reveal a negatively predictive relationship with engaging/spending versus not engaging/spending on loot boxes (i.e. game-related experiences of flow are higher in the nLB cohort). However, once people are engaging/spending, flow becomes positively predictive of increasing spend and risky engagement. One possible explanation is that many loot box containing games are disruptive of flow experiences, i.e. owing to the intrusive nature of loot boxes on the gaming experience. However, once gamers start to actually engage with loot box games, increasing engagement with loot boxes may improve the game experience (and/or lead to greater dissociative phenomena, such as time distortion, escapism, etc.), thus increasing the experience of flow state.

Previous qualitative research has already established that progression is a key motivator in loot box engagement [46], so future qualitative research could specifically investigate flow states in this context. Our results are also consistent with previous distinctions that have been made between positive and negative ‘dark flow’ experiences [38]. It could be argued that flow is a ‘positive’ gaming experience when not associated with loot boxes (i.e. in the non-loot box cohort); while a form of ‘dark flow’ is related to risky loot box engagement within the loot box gaming cohort (i.e. flow driven by potential escape motivations). Again, qualitative research would allow for a richer understanding of how flow can manifest in different gaming scenarios.

Flow was one of the least labile of the non-demographic variables between different model combinations. Nonetheless, the variable that impacted the most on flow was problem video gaming. The apparent overlap between these variables adds further support to notions of ‘dark flow’, suggesting that flow might relate to aspects of problematic gaming behaviour.

### 4.6. Sex

The single predictor models suggested that male sex was somewhat predictive of engaging versus not engaging on loot boxes and with loot box spend, but it was not predictive of increasingly risky engagement. However, the multiple regressions produced somewhat confusing findings. The relationship between sex and engaging with loot boxes disappeared, the association with male sex and increasing spend remained, but now *female* sex became associated with increasingly risky engagement. Along with the other demographic variables, sex had low lability between different model combinations, with minimal disruption from most variables—although problem video gaming (perhaps unsurprisingly) was the most disruptive variable to sex.

The higher prevalence of internet gaming disorder (problem video gaming) among males [52] may partially explain why male sex is predictive of increasing loot box spend in this cohort. The opposite result for RLI (i.e. female sex predictive of increasingly risky engagement in the multiple regression) may be because the bivariate relationship typically seen with sex has shared variance with other variables (e.g. problem video gaming) and when this is accounted for the unshared variance of male sex becomes protective against risky engagement. Further research is required.

### 4.7. Age

The results of earlier pre-registered, bivariate hypotheses for age [19] were somewhat mixed—where age was inversely related to risky loot box engagement, but not to increasing loot box spend. The single predictor models differed in that younger age was somewhat predictive of both risky loot box engagement and loot box spend across both model components. This discrepancy between the bivariate correlations and the single predictor models might be because the Bayesian regressions are able to detect a relationship in the data that the frequentist correlations cannot—or because of added model sensitivity attributable to the negative-binomial response distribution. Regardless, we interpret this as a mixed finding and advise some caution. The result of the multiple regressions revealed that younger age remained predictive both of engaging versus not engaging with loot boxes and of increasingly risky engagement, but not for loot box spend. Across model combinations, age had low lability.

Explanations for why younger age is predictive of risky engagement but not spend is most likely linked to the lower disposable income of younger gamers (as seen in our results [19])—i.e. while younger gamers can still engage riskily with loot boxes, net expenditure is limited by financial constraints.

### 4.8. Income

For income, both the single predictor models and the pre-registered bivariate analyses revealed no association between income and loot box engagement. This finding remained in the multiple regressions, for both loot box engagement and loot box spend (across both model components). Furthermore, income had low lability across models with minimal disruption from other variables, although the largest disruption came from age, which is consistent with our previous observations [19]. These findings are consistent with previous research suggesting no relationship between income and loot box expenditure [27].

### 4.9. Wellbeing and distress

Wellbeing and distress revealed some of the most notable changes in predictiveness between single- and multiple-predictor models. Of the two variables, only distress was associated with participants spending/engaging versus not spending/engaging on loot boxes. Both were associated with increasingly risky loot box engagement. Neither was associated with increasing spend.

In the multiple-predictor models, distress remained a positive predictor of engaging versus not engaging on loot boxes, with positive wellbeing now also becoming predictive. Conversely, distress became non-predictive of increasingly risky loot box engagement, as did wellbeing. The same pattern of results was also seen for loot box spend as the outcome—a finding consistent with an earlier study suggesting no relationship between wellbeing and loot box spend among loot box purchasers [17]. However, note that both variables were associated with spending versus not spending on loot boxes, i.e. a positive association with increasing wellbeing and distress.

Wellbeing and distress both had comparatively high lability across model combinations, with distress generally being the most labile. Unsurprisingly, both of these variables were highly disruptive of each other, illustrating the overlapping (but inverse) relationship between these two psychological constructs. Problem video gaming was also disruptive of distress, highlighting the negative psychological aspect of disordered gaming.

The finding that higher wellbeing and higher distress are both predictive of participants engaging/spending versus not engaging/spending on loot boxes appears contradictory. However, it is possible that there are both psychologically positive and negative aspects of loot box involvement, which could reflect contrasting reasons and motivations. This view is consistent with previous qualitative research showing enhancement motivations related to fun and recreation, in addition to distraction/compulsion motivations related to urges and escapism from life issues [46]. Furthermore, previous research has established that both positive and negative moods can be associated with loot box spending [16], albeit with small effect sizes. For example, loot box engagement may sometimes be a recreational spending linked to higher wellbeing, while at other times being a risky spending decision designed to distract from other problems. Additionally, this finding differs from gambling research, where higher psychological distress is predictive of increased gambling involvement and disordered gambling [53,54]. However, there is little evidence of an equivalent result for higher wellbeing, apart from an equivocal result in a single youth study where positive mood was associated with enhancement motivations for gambling, but not with gambling disorder [55]. These findings might indicate interesting differences between loot boxes and other forms of gambling, although our models showed no relationship (for both wellbeing and distress) with either increasing loot box spend or increasingly risky engagement. Fully understanding these processes is beyond the scope of this study. More research is required.

### 4.10. Limitations and future work

Our sample was limited to a convenience sample of UK adults from the Prolific platform, which may be liable to similar selection biases as other online surveys [56], although there is evidence that it surpasses both MTurk and undergraduate student samples on a number of data quality measures [57,58]. Beyond dataset-related limitations, discussed in more detail in the previous linked paper, the analysis presented here has some further specific limitations. Impulsivity was measured as a unidimensional construct; however, recent research published after our data collection window suggests splitting impulsivity into several sub-factors [13], and future research could measure impulsivity in this way. Further research could also investigate a role for different loot box motivations using the RAFFLE scale [46]. For example, different sub-scales (e.g. social pressure, distraction/compulsion and enhancement) would be interesting to investigate as competing predictors of loot box engagement. There is also debate about flow, including what it is as a psychological construct, how to measure it, and the brain mechanisms this might give rise to it [59–61]. The full complexities of flow may not be fully captured by the 5-item Game Experience Questionnaire scale. A new scale made available after we completed data collection shows potential in capturing some of these complexities [62], but does not measure discriminate between ‘positive’ and ‘dark’ flow, so there may be scope for future scale development work.

Further studies could make further use of longitudinal data collection to investigate the causal direction of the effects we report, building on the first longitudinal study of loot boxes [63]. Additionally, there are several areas where qualitative research would be useful in unpacking the complexities we have discussed—for example understanding the different types of flow that might either protect against or increase engagement with loot boxes.

It is also worth noting that there is evidence that video gamers, when completing the PGSI in surveys like ours, might be referring to their loot box purchasing when completing the PGSI [64]. In our survey, we deployed pre-screening questions for gambling participation, but future studies could specifically prompt participants to not refer to loot boxes before completing the PGSI questionnaire.

Our survey used self-report measures of expenditure. There is previous evidence endorsing self-report approaches in gambling as generally reliable [65], but subsequent evidence has shown participants make estimation errors for both gaming and gambling [24,25,66,67], and there is a need for evidence specifically on loot box expenditure. However, self-report spend data are widely used in research, including our own, because of the difficulties in obtaining account data. There is ongoing debate about whether problem video gaming constitutes a psychological disorder and whether the IGD scale is an appropriate measurement instrument [68–71]. One critique of problem gaming as a formal disorder observes that there are multiple meanings of the term ‘addiction’, and that not all activities characterized as addictive are inherently harmful (i.e. internet gaming disorder might be a ‘mixed blessing’—sometimes harmful; sometimes healthful) [72]. Additionally, the PGSI, IGD and RLI scales have a similar construct and wording. Nonetheless, these are widely used validated scales, and their similarity may be an artefact of such addictive behaviours being underpinned by the same (or similar) cognitive mechanisms. Our results show complex and somewhat nuanced relationships between these measures, suggesting that they are to some extent capturing unique psychological constructs, despite potentially sharing some underlying mechanisms. However, these issues are ultimately challenges for the wider field that are beyond the scope of our current study.

## 5. Conclusion

Our findings reveal that the relationships between various psychological constructs and loot box engagement are more complex than suggested by much previous literature, which have often reported simple bivariate correlations [4–8]. The results challenge notions of a straightforward, direct causal link between loot box engagement and symptoms of problem gambling, instead suggesting that this relationship might be underpinned by shared variance with problem video gaming and gambling-related cognitions, which concomitantly drive risky engagement with video games and loot boxes. Perhaps most notably, the multiple regressions provided no evidence of a relationship with risky loot box engagement, although there was a relationship with increasing spend on loot boxes. The underlying reasons for the surprising result with problem gambling score is elucidated by the lability analysis, revealing how different variables influence each other in the multiple regressions. Here, the lack of association with problem gambling score is primarily explained by disruption from gambling-related cognitions and problem video gaming.

It makes intuitive sense that gambling-related cognitions and problem gambling scores disrupt each other, since both measures capture gambling risks. Gambling-related cognitions encompass factors such as illusion of control, a perceived inability to stop gambling and interpretive control/bias, and it is therefore plausible that such erroneous beliefs may be concomitantly driving risky behaviours in both gambling and loot boxes. Here, there is emerging evidence of directional links between loot boxes and other forms of gambling. For example, a recent longitudinal study found that loot box engagement predicted gambling initiation six months later in a sample of young adult gamers [63]. This is consistent with earlier cross-sectional evidence (using retrospective survey questions) of apparent ‘gateway effects’ from loot boxes into gambling [29]. Such apparent links between loot boxes and traditional gambling require further investigation alongside problem video gaming and gambling-related cognitions.

Similar to gambling, associations between impulsivity and loot box engagement are not observed within our multiple regression, with the most straightforward explanation that impulsive behaviours relevant to loot boxes are largely mediated via problem video gaming. Such a result may help explain equivocal results in the literature [9,10]. However, any assumptions around directions of causality will need to be established via further research.

Our study does not provide any definitive answers around links between loot box engagement and player wellbeing or psychological distress. Instead, the equivocal results in our work and elsewhere [15–17] highlight that higher-level constructs like ‘wellbeing’ and ‘distress’ are influenced by a myriad of other factors (i.e. lifestyle, employment, relationships, etc.), and any relationship with a specific behaviour (such as loot box expenditure) is liable to be swamped by other factors. Nonetheless, when included within our Bayesian multiple regressions, both higher wellbeing and higher psychological distress predicted gamers spending versus not spending on loot boxes—echoing earlier findings that loot box engagement is associated with both positive and negative moods [16]. However, our results showed no relationship between distress or wellbeing, and either increasing loot box spend or increasingly risky loot box engagement.

A novel discovery from this research is the complex relationship between game-related experiences of flow and loot box engagement. Here, experiences of flow were negatively related to the decision to engage with loot boxes, but were positively related to increasing spend and risky behaviour. One possible explanation is that many loot boxes can be intrusive to the gaming experience and feelings of flow, but once gamers start to actually engage with loot box games, increasing loot box engagement may be required to improve the game experience and flow state. Our results may also be evidence of different types of flow, where positive flow experiences protect gamers against stating to engage with loot boxes, while ‘dark flow’ predicts increasingly risky engagement among gamers who already use loot boxes. Establishing the exact nature of possible causal pathways is beyond the pre-registered and exploratory aims of this study, although our variable-by-variable discussion highlights a number of hypotheses that could be investigated in future, particularly around different types of flow states.

Whatever the nature of such relationships, our results suggest that strong relationships exist between risky loot box engagement and other potentially risky activities, such as problem video gaming and gambling-related cognitions (which itself captures cognitive aspects of potentially problematic gambling).

As stated in our previous linked paper, the UK government is adopting an approach of industry self-regulation [73]. However, it is known that industry compliance to features such as odds disclosures and game labelling (through statements such as ‘in-game purchases includes random items') is consistently unsatisfactory [74–76]. Some online storefronts have compliance rate as low as only 7.1% [74], and such measures have doubtful use [77]; being poorly comprehended and largely ignored by adults, children and parents alike. Unless ‘tangible results begin to be seen in the near future’ (as requested by the UKs Department for Digital, Culture, Media & Sport [73, point 32]), then they should ‘not hesitate to consider legislative options… to protect children, young people and adults'. As proposed in our previous paper, such legislation should include a range of consumer protection measures, including enforced age restrictions and customizable spending limits, acting alongside mandatory, clear and upfront labelling and odds disclosures [2].

## Data Availability

Both works have been pre-registered on OSF (https://osf.io/a5nwj), and all underlying data and analysis code is available (https://osf.io/gh634).

## Footnotes

1 Here, there was a minor discrepancy with the RLI ZI component. See the supplementary material on OSF.

2 Again, there was a minor discrepancy with the RLI ZI component. See the supplementary material on OSF.
